# Genetic variation of reef-building polychaete *Sabellaria chandraae* in the south-eastern Arabian Sea based on mitochondrial COI gene sequences 

**DOI:** 10.1080/23802359.2019.1673223

**Published:** 2019-09-30

**Authors:** Periasamy Rengaiyan, Baban Ingole

**Affiliations:** CSIR-National Institute of Oceanography, Goa, India

**Keywords:** Mitochondrial DNA, cytochrome c oxidase subunit I, genetic diversity, West Indian Coastal Current

## Abstract

The honeycomb worm *Sabellaria chandraae* is the most common reef-building polychaete in the intertidal area along the south-eastern Arabian Sea. We used the mitochondrial cytochrome c oxidase subunit I (mtCOI) gene to examine the levels of genetic variation the population. The samples were collected from seven intertidal areas along the south-eastern Arabian Sea. The hierarchical Analysis of Molecular Variance (AMOVA) and conventional population statistics (*F_ST_*) showed a low level of genetic differentiation (*F_ST_* = 0.0864, *p* < .05), indicating no geographical patterning among seven populations. The present results were able to provide a reference for the single stock population along the south-eastern Arabian Sea.

The reef-building worms *Sabellaria chandraae* de Silva, 1961, is broadly distributed along the intertidal zone of the Indian and Sri Lanka coast (Nishi et al. 2010). The habitat selection by larvae and massive construction of reefs in the intertidal rocky area, require the strong flow of water for the transport of the tube-building material and reasons its presents in the west coast of India (Achari 1974). In the Indian coastal system, the boundary currents of Western India Coastal Current (WICC) and Eastern India Coastal Current (EICC) plays an important role in the larval dispersal pattern along with the India coast. The WICC flows towards the equator during the Indian summer monsoon season. That is major recruitment period for many benthic organisms along the Indian coast (Sivadas and Ingole 2016). The benthic polychaetes showed 34% of the species being common between the western and eastern basin which was also confirmed by the genetic (Kumar et al. 2012; Kunal et al. 2013) and larval dispersal studies (George et al. 2011). They are influenced by water circulation pattern, availability of suitable habitat, population fecundity and stochasticity of recruitment success (Dawson et al. 2014). The origins of the reef-building worms yet remain unclear, but molecular studies will finally bring the true evolutionary relationship (Telford 2004). We used the mtCOI gene to investigate the genetic diversity and population variation of 62 *S. chandraae* individual specimens along the ∼1260 km coastline of the south-eastern Arabian Sea between November 2014 and August 2015.

Specimens collected using a hand from the reefs and preserved in 95% ethanol until DNA extraction. The species was identified based on the key characteristic described in Nishi et al. (2010). Preserved specimens are stored at room temperature in the National Institute of Oceanography (CSIR-NIO, Goa, India) (voucher numbers in Table 1). Total genomic DNA was extracted using the commercial QIAamp Tissue Kit (QIAGEN, Valencia, CA). The partial sequences of mtCOI fragments were amplified using the universal primers (Carr et al. 2011). The PCR reactions were carried out in a total volume of 50 μl that consisted of 50–100 ng genomic DNA, 1 × PCR buffer, 2.5 mM MgCl2, 0.2 mM dNTPs, 10 µM primers, and 5U Taq DNA polymerase (Sigma, St. Louis, MO). The temperature profile was as followed: 94 °C for 3 min; 30 cycles of denaturation at 94 °C for 30 s, annealing at 54.5 °C for 30 s and extension at 72 °C for 60 s; followed by a final extension at 72 °C for 5 min. The PCR products were examined on 1% agarose gels, stained with Genecolour^TM^ (Biotium, Hayward, CA), and photographed with transmitted illumination. The PCR-amplified DNA fragments were purified using QIAquick gel purification kit according to manufacturer’s instructions (QIAGEN, Valencia, CA). Sequences were produced using the same primers and determined on an Applied Biosystems (ABI, Foster City, CA) 3730xl automated DNA sequencer, following the standard cycle sequencing protocol. The sequences were submitted to NCBI GenBank. The mtCOI gene sequences were aligned using CLUSTAL W (Thompson et al. 1994) implemented in BIOEDIT (Hall 1999). Hierarchical analysis of molecular variance (AMOVA) was used for separating of genetic variance within and among the localities and between the two regions (Northern and Southern). Genetic differentiation was examined by means of pairwise *F_ST_* values, using 10,000 permutations to determine significance in Arlequin 3.5 (Excoffier and Lischer 2010).

**Table 1.** Details of nucleotide and haplotyde diversity showed in the COI gene sequences of *Sabellaria chandraae* along the south-eastern Arabian Sea.

Geographic region	No. of sequences	No. of haplotypes	Haplotype diversity, *h*	Nucleotide diversity, *π*	Lat_Long	Specimen voucher numbers	Genbank accession numbers
Ganapatipule	7	2	0.25	0.0005	17.08 N, 73.16 E	GP0348-GP0355	KX525531-KX525538
Devgad	8	4	0.694	0.001	15.41 N, 73.41 E	DP0339-DP0347	KX525522-KX525530
Vengurla	9	2	0.2	0.0007	15.49 N, 73.38 E	VP0393-VP0402	KX525576-KX525585
Arambol	5	1	0.333	0.0005	15.41 N, 73.41 E	AP0333-AP0338	KX525516-KX525521
Palolem	9	1	0	0	15.00 N, 74.01 E	PP0383-PP0392	KX525566-KX525575
Mangaluruu	10	1	0	0	13.11 N, 74.44 E	MP0365-MP0375	KX525548-KX525558
Kanyakumari	8	1	0	0	11.52 N, 75.21 E	KP0376-KP0384	KX525539-KX525547

The Haplotype (*h*) and nucleotide (*π*) diversities were estimated using DnaSPv5 (Nei 1987). Total haplotype diversity values of mtCOI gene being 0.21. Total nucleotide diversity of mtCOI gene sequence was 0.0005 (Table 1). The low genetic variation in *S. chandraae* population is consistent with the boundary currents of WICC play an important role in the larval dispersal pattern along the west of coast India. The accurate test of population differentiation (non-differentiation exact P values) showed no significant differences among seven samples. The AMOVA results of *S. chandraae* shows within populations (*F_CT_* = 0.036; *p* > .795) and among populations (*F_ST_* = 0.086; *p* > .1,) between north and south populations (Table 2). The existence of genetic variation confirmed a single stock population of *S. chandraae* species along the ∼1260 km the west coast of India. Since, the distribution of the species depends the existence of historical factors, reversing WICC, lacking an oceanic barrier to the larval movement among study areas were further confirmed by the low values of *F_ST_*.

**Table 2.** Analysis of molecular variance (AMOVA) of *Sabellaria chandraae* between the populations.

Source of variation	*df*	Sum of squares	Percentage of variation
Among groups	1	0.095	3.68
Among populations within groups	5	1.058	12.32
Within populations	56	5.386	91.36
Total	62	6.539	100
